# Cyclodextrin-Polypyrrole Coatings of Scaffolds for Tissue Engineering

**DOI:** 10.3390/polym11030459

**Published:** 2019-03-11

**Authors:** Jan Lukášek, Šárka Hauzerová, Kristýna Havlíčková, Kateřina Strnadová, Karel Mašek, Martin Stuchlík, Ivan Stibor, Věra Jenčová, Michal Řezanka

**Affiliations:** 1 Department of Nanomaterials in Natural Science, Institute for Nanomaterials, Advanced Technologies and Innovation, Technical University of Liberec, Studentská 1402/2, 461 17 Liberec, Czech Republic; jan.lukasek@tul.cz (J.L.); martin.stuchlik@tul.cz (M.S.); ivan.stibor@tul.cz (I.S.); 2 Institute of New Technologies and Applied Informatics, Faculty of Mechatronics, Informatics and Interdisciplinary Studies, Technical University of Liberec, Studentská 1402/2, 461 17 Liberec, Czech Republic; 3 Department of Nonwovens and Nanofibrous Materials, Faculty of Textile Engineering, Technical University of Liberec, Studentská 1402/2, 461 17 Liberec, Czech Republic; sarka.hauzerova@tul.cz (Š.H.); kristyna.havlickova@tul.cz (K.H.); katerina.strnadova@tul.cz (K.S.); 4 Department of Surface and Plasma Science, Faculty of Mathematics and Physics, Charles University, V Holešovičkách 2, 180 00 Prague 8, Czech Republic; karel.masek@mff.cuni.cz; 5 Department of Chemistry, Faculty of Science, Humanities and Education, Technical University of Liberec, Studentská 1402/2, 461 17 Liberec, Czech Republic; vera.jencova@tul.cz

**Keywords:** cyclodextrin, pyrrole, polypyrrole, polycaprolactone, functionalisation, microfibres, tissue engineering, scaffold

## Abstract

Polypyrrole is one of the most investigated conductive polymers used for tissue engineering applications because of its advantageous properties and the ability to promote different cell types’ adhesion and proliferation. Together with β-cyclodextrin, which is capable of accommodating helpful biomolecules in its cavity, it would make a perfect couple for use as a scaffold for tissue engineering. Such scaffolds were prepared by the polymerisation of 6-(pyrrol-3-yl)hexanoic acid on polycaprolactone microfibres with subsequent attachment of β-cyclodextrin on the polypyrrole layer. The materials were deeply characterised by several physical and spectroscopic techniques. Testing of the cyclodextrin enriched composite scaffold revealed its better performance in in vitro experiments compared with pristine polycaprolactone or polypyrrole covered polycaprolactone scaffolds.

## 1. Introduction

Tissue engineering [1,2] is a very complex science discipline that aims to develop methods for tissue renewal and, thus, tries to imitate or improve the processes that take place everywhere in nature. One promising material for fabricating scaffolds, which are used for achieving this goal, is polypyrrole (PPy) [3]. Pyrrole—a five-membered heterocycle occurring in natural compounds—is used for PPy preparation by oxidative polymerisation. PPy is one of the most investigated among conductive polymers used for tissue engineering applications because of its high stability, biocompatibility, simple preparation, structural tunability, and its ability to promote the adhesion and proliferation of different cell types [3,4,5]. Moreover, tests of PPy powder extracts for acute and subacute toxicity, haemolysis, and cell viability showed no negative results [6].

Cyclodextrins (CDs) are naturally occurring glucose-based cyclic oligosaccharides. Among them, β-CD (possessing seven glucose units in the cycle) has a prominent position because of its easy availability. Cyclodextrins are best known for their ability to accommodate lipophilic guests in their cavity in an aquatic environment. This feature also plays a key role in their use in tissue engineering scaffolds, where CDs have proven to be beneficial [7,8,9]. The β-CD functionalised polymer could be used for the fabrication of supramolecular 3D scaffolds [10]. It has also been shown that β-cyclodextrin (β-CD) functionalised scaffolds promote performance in tissue engineering [11] by sequestration of growth factors [12], by an increase in oxygen concentrations in tissue engineered constructs [13], and by the regulation of collagen self-assembly [14]. Moreover, β-CD functionalised scaffolds are able to release drugs during cell culturing [15,16,17].

Based on the abovementioned findings, CD-PPy covered scaffolds would, thus, open the way for a versatile material, which can bind biomolecules on its surface (without the need of covalent bond attachment), thereby, enhancing cell adhesion, proliferation, or the possession of other desired features. Such a material aspires to have many advantages, including better attachment of biomolecules, easy modification of surfaces, the stability of the included biomolecules, and suitability as a scaffold for nerve tissue engineering. To the best of our knowledge, there are no known CD modified polypyrrole scaffolds used for tissue engineering.

Herein, we wish to report the preparation of microfibrous polycaprolactone (PCL) scaffolds functionalised with poly [6-(pyrrol-3-yl)hexanoic acid] (PPyHA) layer bearing β-CD units. The testing of a cyclodextrin enriched composite scaffold revealed its better performance in in vitro experiments compared to pristine PCL or PPyHA covered PCL scaffolds.

## 2. Materials and Methods

### 2.1. Synthesis of Pyrrole Monomer

Nuclear magnetic resonance (NMR) spectra were recorded using JEOL JNM-ECZR at 500 MHz for ^1^H and at 126 MHz for ^13^C. High-resolution mass spectrometry (HRMS) was measured using LTQ-Orbitrap Velos (APCI^+^). Silica gel 60 (Merck, Darmstadt, Germany) was used for column chromatography.

An oven-dried flask was charged with 6-[(1-triisopropylsilyl)pyrrol-3-yl)]hexanoic acid (2 g, 5.92 mmol) [18], and evacuated and refilled with argon three times. Dry tetrahydrofurane (THF, 29 mL) was added using a cannula, and the reaction mixture was cooled to 0 °C. The reaction mixture was then treated with 1.05 eq. (6.22 mL, 1 M in THF) of tetrabutylammonium fluoride (TBAF) at 0 °C and stirred for 1 h. All volatiles were then evaporated under reduced pressure, and a semi-solid residue was purified by column chromatography on silica gel with a gradient mobile phase (CHCl_3_:MeOH 40:1, 20:1, 5:1). 6-(pyrrol-3-yl)hexanoic acid (934 mg) was isolated as a grey solid in 87% yield.

^1^H NMR (500 MHz, CDCl_3_) *δ* 9.24 (br s, 1H), 6.36 (ddd, *J* = 2.6, 2.0, 0.2 Hz, 1H), 6.23 (ddd, *J* = 2.5, 2.0, 1.6, 0.8 Hz, 1H), 5.70 (ddd, *J* = 2.7, 1.6, 0.5 Hz, 1H), 2.17 (t, *J* = 7.6 Hz, 2H), 1.97 (t, *J* = 7.6 Hz, 2H), 1.34 (quint, *J* = 7.3 Hz, 2H), 1.29 (quint, *J* = 7.4 Hz, 2H), 1.10 (quint, *J* = 7.7 Hz, 2H) (for more details see Figure S1); ^13^C NMR (126 MHz, CDCl_3_/MeOD) *δ* 175.6, 122.9, 116.8, 114.2, 107.2, 33.6, 30.4, 28.4, 26.2, 24.2 (for more details see Figure S2); HRMS (APCI^+^): calcd for C_10_H_15_NO_2_ 182.11756 [(M + H)^+^)]; found *m*/*z* 182.11757 [(M + H)^+^)].

### 2.2. Preparation of Scaffolds and Their Characterisation

#### 2.2.1. Preparation of Electrospun PCL Fibres

Polycaprolactone (PCL) with a molecular weight of 80,000 (Merck) was electrospun. The spinning was carried out from a 10% (*w*/*w*) polymer solution in a solvent system composed of chloroform, ethanol and acetic acid (8/1/1, *v*/*v*/*v*;). The planar fibrous layer was prepared using a needleless electrospinning technology and carried out by Nanospider^TM^ 1WS500U (Elmarco, Liberec, Czech Republic). The 0.2 mm string and 0.5 mm slots were used. The forming fibres were collected on a spun bond layer placed 16 cm above the string and rolled by a speed of 10 mm/min. The applied voltages of −10 kV and +35 kV were used in the collector and the string, respectively. The temperature was 20 °C and relative humidity was 36–41%. For a morphology evaluation, samples were observed by scanning electron microscope (SEM) using a Tescan Vega3 SB Easy Probe. The samples were sputter coated with gold (7 nm) before analysis. The morphological analysis of the images from the SEM was performed by NIS Elements software (Nikon, Tokyo, Japan). The fibre diameter (1.13 ± 0.36 μm) was evaluated from 100 measurements.

Specific surface analysis (BET) was performed on the Autosorb iQ, Quantachrome instrument in a standard mode. The fibrous sample (291.8 mg) was put into a 12 mm glass BET cell and degassed for 24 h at 40 °C before measurement. Krypton was used for the analysis, and the data were processed using ASiQwin software (Figure S3). The resulting specific surface area of the analysed sample was 2.747 m^2^/g.

#### 2.2.2. Deposition of PPyHA Layer onto PCL

A glass vial containing 6-(pyrrole-3-yl)hexanoic acid (18 mg, 0.1 mmol) was charged with 3.4 mL of methanol. The PCL sheet (area weigh 5 mg/cm^2^) was cut into 35 circles (diameter 0.9 cm) and the samples were immersed in the monomer solution for 60 min. 2.4 Eq. of FeCl_3_.6H_2_O (65 mg, 0.24 mmol) in 0.6 mL of methanol was then added in one portion to reach the pyrrole monomer concentration of 2.5 mM. The reaction was shaken (150 rpm) at room temperature for 12 h while the samples became black. This indicated the formation of insoluble polypyrrole. All of the samples were rinsed thoroughly with methanol and sonicated five times in another methanol for 5 min. Finally, the samples were air and vacuum dried before being stored in a desiccator.

X-ray photoelectron spectroscopy (XPS) measurements of the prepared PCL-PPyHA sample were performed in an ultra-high vacuum chamber with a base pressure below 10^−7^ Pa. The spectra were taken at a normal emission angle using a Specs XR50 x-ray source with Al and Mg anodes (h*υ* = 1486.6 eV for Al K_α_ and h*υ* = 1253.6 eV for Mg K_α_ radiation, respectively) and a VSW HA100 hemispherical analyser with multi-channel detection. The samples were measured in an “as received” state without any cleaning of the surface. The chemical state of the samples was investigated by fitting C 1 s, O 1 s and N 1 s core level spectra in the KolXPD software. Spectral lines were represented by pseudo-Voigt functions, and a Shirley-type background was subtracted from the spectra.

Moreover, a Nicolet^TM^ iZ^TM^10 FT-IR Spectrometer (Thermo Scientific^TM^, Waltham, MA, USA) and a Q500 thermogravimetric analyser (TA Instruments, New Castle, DE, USA) were used for further characterisation of the PCL-PPyHA scaffolds. The sample for thermogravimetric analysis (TGA) was placed on a platinum pan, and the thermal properties were analysed using a nitrogen atmosphere with a flow rate of 60 mL/min. The analysis ran from room temperature to 650 °C with a gradient of 10 °C/min. Temperature and weight loss were not correlated, and TA Universal Analysis software was used for data processing.

The electrical sheet resistance of the prepared scaffolds was examined by a standard two-point technique using the KEITHLEY 487 picoampermeter [19]. The contacts were prepared by the cathode sputtering of Au at a current of 40 mA and a sputtering time of 200 s. Under the conditions described, their thickness was approximately 75 nm. The measurements were carried out under reduced pressure (10 Pa) to eliminate the influence of air humidity. The electrical sheet resistance of the PCL-PPyHA scaffold was determined to be 2.22 ± 0.02 TΩ.

#### 2.2.3. Immobilisation of CD onto PPyHA Modified PCL

PCL-PPyHA samples from the previous experiment were sonicated in a 2-(*N*-morpholino)ethanesulfonic acid (MES) buffer (10 mM, pH 6) until they were completely soaked with the solution. The samples were then transferred to 12 mL of fresh MES buffer. *N*-(3-Dimethylaminopropyl)-*N*′-ethylcarbodiimide (EDC, 200 mg, 1.04 mmol) was dissolved together with *N*-hydroxysuccinimide (NHS, 300 mg, 2.6 mmol) in 8 mL of MES buffer and the solution was added in one portion to the reaction mixture. The reaction mixture was gently shaken at 150 rpm for 3 h at room temperature. After activation of carboxy groups, the solution was removed, and 20 mL of a fresh MES buffer with dissolved 6^A^-deoxy-6^A^-amino-β-CD (47.2 mg, 41.6 μmol) was added. The functionalisation proceeded for 12 h at room temperature before the buffer solution was removed. The samples were rinsed with distilled water, sonicated 6 times for 5 min in water and then allowed to air dry. Finally, all of the samples were dried under a vacuum and stored in a desiccator.

A series of rhodamine B solutions in methanol was prepared as standard, and the fluorescence was measured in a quartz cuvette at a wavelength of 545 nm using a TECAN Spark spectrometer. PCL-PPyHA and PCL-PPyHA-CD samples (52 mg) were soaked in 4 mL of MeOH with rhodamine B (10^−7^ M) for 30 min, and the fluorescence was measured.

### 2.3. Cell Culturing

Samples of planar fibrous materials were tested in vitro using normal human dermal fibroblasts (NHDF). The cell line was obtained from ATCC (LGC Standards, Lomianki, Poland). The cells were cultured in a Fibroblast Growth Medium-2 BulletKit^TM^ (FGM-2, Lonza, Basel, Switzerland) at 37 °C and 5% CO_2_. The materials were cut into round samples (*d* = 10 mm), sterilised by 70% ethanol for 30 min and washed several times in phosphate-buffered saline (PBS, pH 7.4) before cell seeding. NHDF cells in passage 8 were seeded on the surface of the material in a concentration of 2000 cells/well. Evaluation of the material–cell interaction was performed after 1, 3, 7 and 14 days of incubation. Cell viability and proliferation were measured by the cell counting test (cck-8, Merck). 250 µL of 10% cck-8 solution (in FGM-2 medium) were added to each sample and incubated for 3 h at 37 °C and 5% CO_2_. The absorbance of the solution after incubation was measured at 450 nm with the reference wavelength at 650 nm.

Visualisation of cells was carried out by fluorescence staining of fibroblasts. The samples were washed two times in PBS (pH 7.4), then fixed with 2.5% glutaraldehyde in PBS (30 min at 4 °C). After fixation, the samples were rinsed in PBS, then stained with 4′,6-diamidino-2-phenylindole (DAPI, Merck) together with fluorescein isothiocyanate labelled phalloidin (phalloidin-FITC) (Merck). The cells were treated in 0.1% Triton (Merck) for permeabilization, and the samples were washed with PBS and incubated with phalloidin-FITC (1 mg/mL stock solution diluted 1:1000 in PBS) for 30 min at room temperature (RT). Then the samples were washed with PBS and incubated with DAPI (5 min, RT). Finally, the samples were rinsed with PBS and analysed by a fluorescent microscope (NICON Eclipse Ti-e).

Multiple comparison pairs were analysed using a one-way ANOVA with Tukey post-hoc test. Significance was defined at a level of *p* < 0.05. For all conditions, *n* = 4 was used for analysis. All values are reported as mean +/− standard deviation.

### 2.4. Analysis of Protein Adsorption to Scaffolds

Interactions of bovine serum albumin (BSA) protein (Merck) with the prepared materials were tested with a BSA solution (50 mg/mL, in PBS with pH 7.4). Round samples (diameter of 1.6 cm, 10 mg) were incubated in 1 mL of BSA solution for 1 h (37 °C, gentle agitation). The samples were then washed twice in PBS followed by desorption of proteins. Desorption was carried out in 1 mL of PBS solution containing 1% sodium dodecyl sulphate (SDS) (Merck), and the samples were gently agitated for 1 h at RT. The protein solutions after desorption were analysed by electrophoresis (SDS-PAGE, Bio Rad) and by liquid chromatography. The experiment was carried out in quadruplicate. For SDS-PAGE, 10 µL of each sample were diluted (1:1) in a loading buffer (0.15 M Tris.HCl, pH 6.8, 30% glycerol (*v*/*v*)), 15% β-mercaptoethanol (*v*/*v*), 1% SDS (*w*/*v*), 0,01% bromophenol blue (*w*/*v*)). Twenty microlitres of the prepared samples were run using 10% gel (2 h, 110 V) with a wide-range molecular weight marker (Protein wide range MW Marker, Amresco, Solon, OH, USA). Subsequently, gels were stained with Coomassie blue solution (2.5% Coomassie brilliant blue R-250 (*w*/*v*) in 45% methanol (*v*/*v*) and 10% acetic acid (*v*/*v*)) and were evaluated after being washed several times in a destaining solution (45% methanol (*v*/*v*) and 10% acetic acid (*v*/*v*)). For the chromatography analysis, 200 µL of the samples were diluted in 1 mL of a solution of 100 mM NaH_2_PO_4_ (pH 6.8), 100 mM NaCl and 0.03% NaN_3_. The same solution was used as the mobile phase during the analysis. The Dionex Ultimate 3000 system with a single Phenomenex Yarra SEC-3000 column (3 µm particle diameter, 300 mm length and 4.6 mm inner diameter) and UV detection (DAD 3000 detector) at 279 nm were used.

## 3. Results and Discussion

### 3.1. Preparation of Scaffolds and Their Characterisation

**Figure 1:**
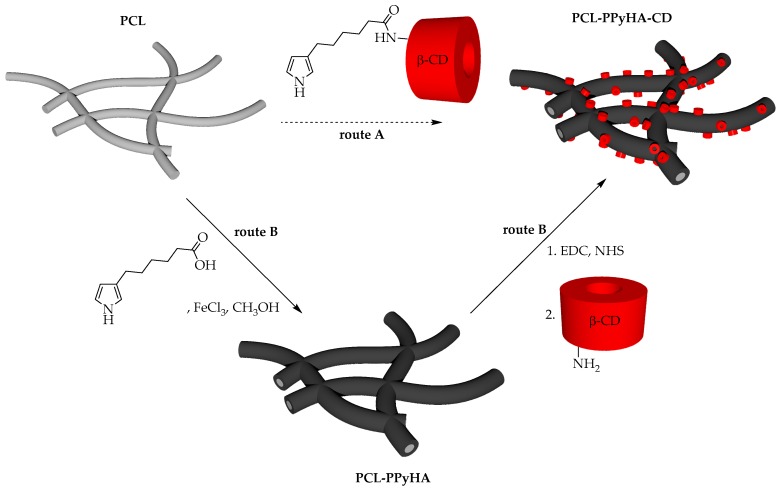
General routes for the preparation of poly [6-(pyrrol-3-yl)hexanoic acid]-cyclodextrins (PPyHA-CD) modified polycaprolactone (PCL) scaffolds. (**Route A**) direct polymerisation of pyrrole-CD conjugate onto PCL fibres. (**Route B**) stepwise functionalisation—polymerisation of 6-(pyrrol-3-yl)hexanoic acid and subsequent coupling with amino derivative of β-cyclodextrin (β-CD). Image not to scale.

The general route A (Figure 1) was followed for preparation of the PPyHA-CD modified scaffold with an electrospun PCL microfibrous platform as a starting material. First, *N*-(6^A^-deoxy-β-cyclodextrin-6^A^-yl)-6-(1*H*-pyrrol-3-yl)hexanamide (Figure 1) was synthesised [18] and used for polymerisation with ferric chloride as an oxidation agent [20]. Surprisingly, the polymerisation did not proceed either under the given reaction conditions or under other ones (using elevated temperature, different oxidizing agents or higher concentration of reagents) published elsewhere [21,22]. Such low reactivity is probably caused by the steric demands of bulky β-CD macrocycle, which blocks α-positions of the pyrrole ring. Thus, an alternative functionalisation sequence (route B, Figure 1) was proposed. 6-(pyrrol-3-yl)hexanoic acid was synthesised starting from its triisopropyl protected analogue [18] by simple cleavage of the protecting group using TBAF in THF at RT. The target pyrrole monomer was purified using column chromatography and isolated in 87% yield.

**Figure 2:**
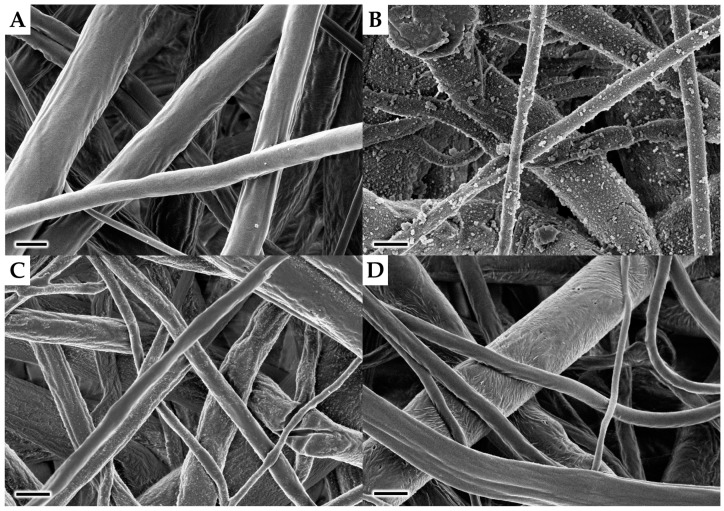
Scanning electron microscope (SEM) images of (**a**) PCL; and polycaprolactone-poly [6-(pyrrol-3-yl)hexanoic acid] (PCL-PPyHA) scaffolds prepared using (**b**) 60% methanol in water (*v*/*v*); (**c**) 95% methanol in water (*v*/*v*); and (**d**) 100% methanol. Scale bar 1 μm.

Based on our experience with polypyrrole chemistry the choice of solvent was considered as one of the most critical parameters for obtaining a smooth polymer layer. The native pyrrole polymerises rapidly in water but slowly in alcoholic solvents due to deactivation of FeCl_3_ as an oxidizing agent [23]. However, the hydrophobic character of the PCL scaffold does not allow the use of pure water as a solvent. Thus, 60% methanol in water (*v*/*v*) was used instead to ensure proper wetting of the PCL fibres and also to keep the polymerisation rate sufficient. As can be seen in Figure 2b, the resulting PPyHA coating on the PCL fibrous matrix had several microscopic defects compared to pristine PCL (Figure 2a). Therefore, subsequent experiments were performed using a different methanol/water ratio (Figure 2c,d). Surprisingly, the polymerisation rate of the pyrrole derivative with the carboxylic group was sufficient even in pure methanol at room temperature, whereas pyrrole alone polymerises very slowly under these conditions [23]. Thanks to the unexpected reactivity and superior wettability of the PCL scaffold in methanol, a smooth layer of PPyHA without any structural defects was achieved (Figure 2d). Moreover, the pending side chains with terminal carboxylic groups represent a binding place for the final β-CD connection.

**Figure 3:**
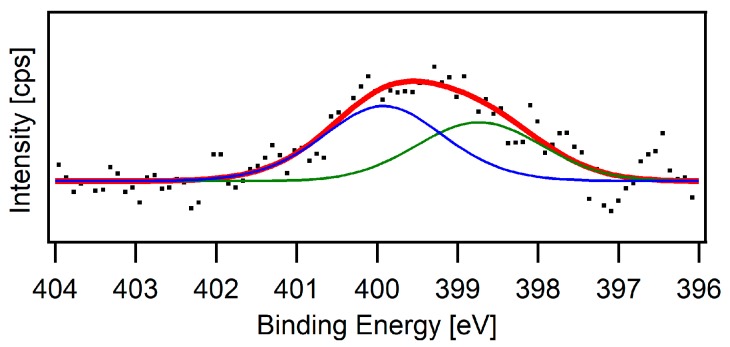
The X-ray photoelectron spectroscopy (XPS) spectrum of N 1 s core level (Mg K*_α_* radiation) of the PCL-PPyHA sample (red). Blue and green lines represent two binding modes of the nitrogen atom.

A number of analytical methods were used for confirmation of the successful deposition of the PPyHA film. Although the characterisation of thin layers on a flat metal surface can be easily studied by XPS [24], the analysis is far more complicated on fibrous scaffolds. PCL has a low melting point (60 °C) and, therefore, cannot be purged by energy-rich argon ions. Moreover, the penetration depth of the radiation is higher in organic materials, and, thus, the overall signal is a combination of the PCL matrix and the thin PPyHA layer on the surface. The spectra of oxygen and carbon were accompanied by surface impurities, and the elemental composition could not be quantified. However, successful coverage of the fibre surface was confirmed by the nitrogen spectrum (Figure 3). The nitrogen signal of the PCL-PPyHA sample is a combination of two out of four possibilities of how the nitrogen atom could be bonded in PPy [25,26]. The other two forms are not visible due to their low intensities.

**Figure 4:**
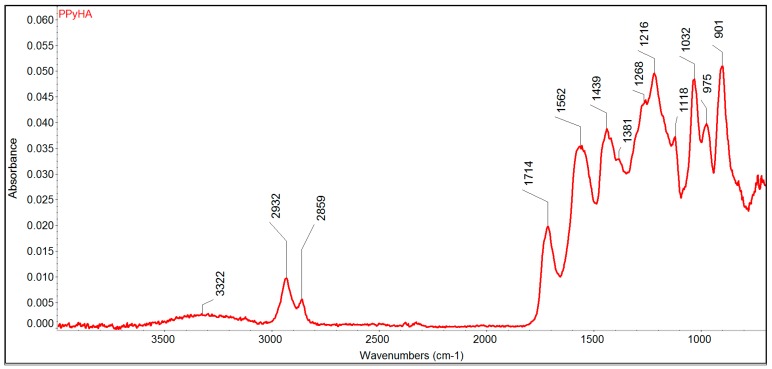
Infrared (IR) spectrum of the resulting PPyHA powder after washing off the PCL.

Next, infrared (IR) spectroscopy was used to confirm the presence of functional groups in the PCL-PPyHA substrate. However, due to the very thin layer of PPyHA, it was not possible to measure it directly. Therefore, the PCL was washed out by CHCl_3_, and the structure of the resulting PPyHA powder was analysed (Figure 4). A broad valence vibration of carboxylic O–H group between 3500 and 3050 cm^−1^ and the characteristic strong symmetrical stretching vibration of C=O group at 1714 cm^−1^ confirmed the presence of carboxylic acid on the polymer structure. Furthermore, the symmetric, as well as asymmetric vibrations of the corresponding alkyl –CH_2_– linker at 2932 and 2859 cm^−1^, can be found as well.

**Figure 5:**
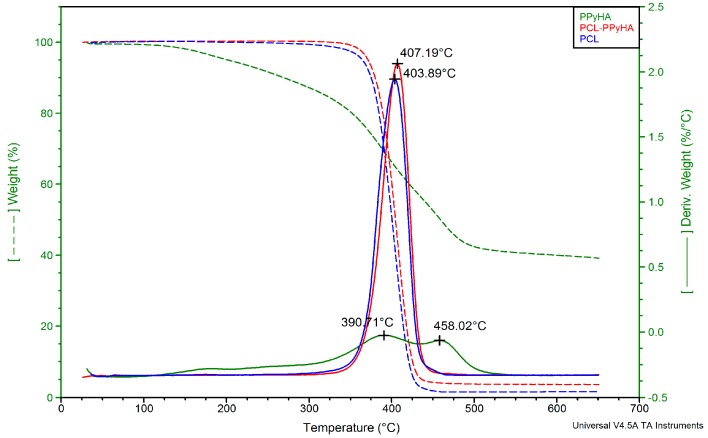
TGA curves and their derivatives (dashed) of PCL (blue), PCL-PPyHA (red) and PPyHA powder after washing off the PCL (green).

TGA was used for further characterisation of the deposited PPyHA layer (Figure 5). The decomposition profile was similar for both PCL and PCL-PPyHA because the PPyHA layer is very thin and does not influence the thermal properties of the matrix. Nevertheless, valuable information about the quantity of deposited polypyrrole could be deducted from the residual weight. While pristine PCL was decomposed nearly entirely (98.3%), the residual weight of powdered PPyHA was 38.2%. It is, therefore, possible to calculate the percentage of PPyHA on the PCL surface. The percentage of deposited PPyHA was 5.2% (*w*/*w*), which is in good agreement with our previously published results [27]. Moreover, such an analysis represents a fast and robust method for confirmation and also quantification of deposited polypyrrole between similar experiments.

To prepare the final PCL-PPyHA-CD scaffold, commonly used 6^A^-deoxy-6^A^-amino-β-cyclodextrin [28,29] was conjugated onto a PCL-PPyHA scaffold with the aid of carbodiimide chemistry. First, carboxylic groups on the surface were activated with EDC/NHS in an MES buffer (pH 6). Then, the solution was replaced with a fresh buffer containing a dissolved β-CD amino derivative, and the scaffold was allowed to incubate overnight.

The maximum theoretical amount of CDs attached to the fibrous surface was estimated to be 0.25% (*w*/*w*). This calculation was based on the specific surface area of the fibres, the outer diameter of CDs, and the hexagonal packing arrangement with the highest possible density of molecules. Due to such a low relative number of CDs on the surface, the abovementioned methods used for characterisation of the PCL-PPyHA scaffold gave the same results for PCL-PPyHA-CD. Therefore, the amount of immobilised CD was measured indirectly by fluorescence labelling. The interaction of CDs with rhodamine B was exploited using the known ability of CDs to form inclusion complexes with various molecules. The PCL-PPyHA material was incubated with rhodamine B, and the results were used as a blank to exclude an interaction with the PPyHA layer. However, due to a low overall number of CDs on the surface of the PCL-PPyHA-CD sample, the difference was negligible. The experimental error could, therefore, be very high, and the amount of deposited CDs could not be determined exactly, even though, the results from cell culturing, protein adsorption (see below) and change in the electrical sheet resistance to 13.81 ± 0.31 TΩ suggests successful functionalisation of the PCL-PPyHA scaffold with CDs.

### 3.2. Cell Culturing and Protein Adsorption to the Scaffolds

**Figure 6:**
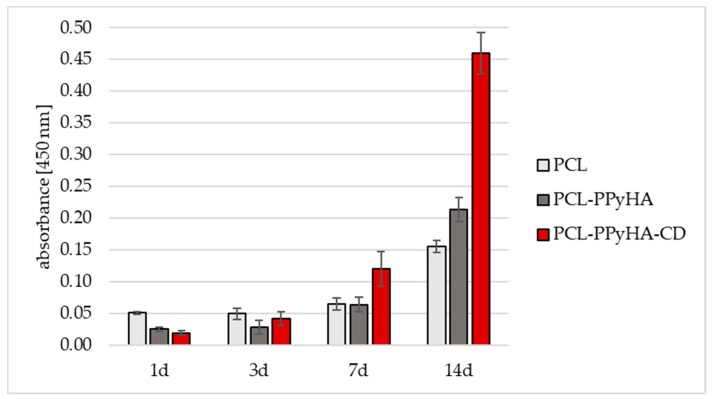
Cell viability analysis. Proliferation of normal human dermal fibroblasts (NHDF) on prepared scaffolds (PCL, PCL-PPyHA, PCL-PPyHA-CD) after 1, 3, 7 and 14 days.

All three prepared scaffolds (PCL, PCL-PPyHA and PCL-PPyHA-CD, Figure 1) were subjected to an in vitro experiment with NHDF cells. The MTT (3-(4,5-dimethylthiazol-2-yl)-2,5-diphenyltetrazolium bromide) cell viability assay together with fluorescence analysis was conducted on days 1, 3, 7 and 14. As shown in Figure 6, the performance of both functionalised materials was slightly worse than pristine PCL on testing days 1 and 3. However, there was a significant increase in cell activity for both samples compared to PCL. The cell viability was significantly higher compared to PCL on day 14 (PCL vs. PCL-PPyHA and PCL vs. PCL-PPyHA-CD; *p* < 0.05). Cells were well spread across the fibrous scaffold with no visible defects (Figures S4 and S5). The introduction of the thin carboxylic acid modified PPyHA layer onto the PCL surface helped cell proliferation and growth, which is in accordance with the abovementioned findings [3,4,5]. Moreover, the immobilised CDs work synergistically with PPyHA and dramatically increase cell proliferation.

**Figure 7:**
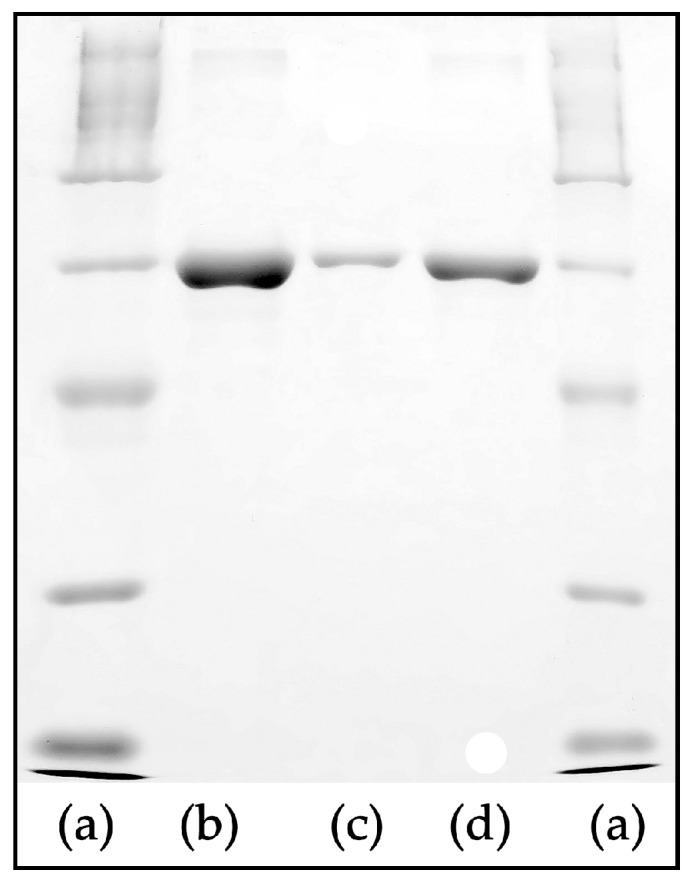
Sodium dodecyl sulphate electrophoresis (SDS-PAGE) results of adsorbed bovine serum albumin (BSA) onto the scaffolds. (**a**) marker; (**b**) PCL; (**c**) PCL-PPyHA; (**d**) PCL-PPyHA-CD.

In general, the adsorption of proteins significantly influences the properties of the materials giving them biological identity [30,31]. Therefore, the protein adsorption was measured to find out if the CD decorated scaffold is capable of accommodating helpful biomolecules. Scaffold samples (10 mg) were used for the adsorption experiment with a model BSA protein, whose concentration was quantified by SDS-PAGE and liquid chromatography after desorption. SDS-PAGE results revealed that the presence of CDs on the surface of the fibrous material leads to a greater degree of protein adsorption compared to the PCL-PPyHA scaffold (Figure 7). However, the pristine PCL scaffold adsorbs far more BSA than the two others. These results were confirmed by liquid chromatography, which showed that the amount of BSA adsorbed onto the materials was 67 ± 9 μg/mg of the PCL sample, 8 ± 3 μg/mg of the PCL-PPyHA sample and 21 ± 8 μg/mg of the PCL-PPyHA-CD sample. In the case of the PCL scaffolds, the best results for protein adsorption are in direct contradiction to the worst results from the cell viability analysis. It is generally known [32] that if the material is too hydrophobic, the proteins could be adsorbed in high amounts, but in a denatured state [33], causing the surface to be less attractive for the cells. The results suggest that BSA is well adsorbed to PCL, but in distorted conformation [34] unsuitable for proper interaction with membrane receptors. However, PCL-PPyHA and PCL-PPyHA-CD scaffolds are less covered, but with native proteins.

## 4. Conclusions

A general platform for functionalisation of non-woven polymers for tissue engineering has been developed. An unexpected high polymerisation rate of a newly synthesised pyrrole monomer—6-(pyrrol-3-yl)hexanoic acid—in methanol was used as an advantage for the preparation of a smooth layer on the PCL fibrous matrix. Deposited polypyrrole bearing hexanoic acid was characterised by physical and spectroscopic techniques to verify its material structure. The surface pending carboxylic groups was subsequently used for conjugation of β-CD via an amide bond. Finally, the biological in vitro experiment revealed that our composite PCL-PPyHA-CD material is not only biocompatible but also greatly improves cell-material interaction. These results will, thus, serve as a cornerstone for future research focused on the design of bioinspired conductive polymers for neural tissue regeneration.

## Supplementary Materials

The following are available online at https://www.mdpi.com/2073-4360/11/3/459/s1, Figure S1: ^1^H NMR spectrum (CDCl_3_/MeOD, 298 K, 500 MHz), Figure S2: ^13^C NMR spectrum (CDCl_3_/MeOD, 298 K, 126 MHz), Figure S3: Multi-point BET plot, Figure S4: Fluorescent microscopy of cells seeded on PCL, PCL-PPyHA and PCL-PPyHA-CD scaffolds on days 1, 3, 7 and 14 after cell seeding, and Figure S5: SEM images of the PCL, PCL-PPyHA and PCL-PPyHA-CD scaffolds on days 1 and 14 after cell seeding.
