# Supplementary material for: Cyclodextrin-Polypyrrole Coatings of Scaffolds for Tissue Engineering
Source: Polymers (Basel). 2019 Mar 11;11(3):459. doi: 10.3390/polym11030459 (PMC6473528; doi:10.3390/polym11030459)
Supplement: Supplementary file 1 [file polymers-11-00459-s001.pdf]

Supplementary Materials for

Cyclodextrin-polypyrrole coatings of scaffolds for tissue engineering

Jan Lukášek [1,2], Šárka Hauzerová [3], Kristýna Havlíčková [3], Kateřina Strnadová [3], Karel Mašek [4], Martin Stuchlík [1], Ivan Stibor [1], Věra Jenčová [5] and Michal Řezanka [1,*]

[1] Department of Nanomaterials in Natural Science, Institute for Nanomaterials, Advanced Technologies and Innovation, Technical University of Liberec, Studentská 1402/2, 461 17, Liberec, Czech Republic; jan.lukasek@tul.cz (J.L.); martin.stuchlik@tul.cz (M.S.); ivan.stibor@tul.cz (I.S.)

[2] Institute of New Technologies and Applied Informatics, Faculty of Mechatronics, Informatics and Interdisciplinary Studies, Technical University of Liberec, Studentská 1402/2, 461 17, Liberec, Czech Republic

[3] Department of Nonwovens and Nanofibrous Materials, Faculty of Textile Engineering, Technical University of Liberec, Studentská 1402/2, 461 17, Liberec, Czech Republic; sarka.hauzerova@tul.cz (Š.H.); kristyna.havlickova@tul.cz (K.H.); katerina.strnadova@tul.cz (K.S.)

[4] Department of Surface and Plasma Science, Faculty of Mathematics and Physics, Charles University, V Holešovičkách 2, 180 00, Prague 8, Czech Republic; karel.masek@mff.cuni.cz (K.M.)

[5] Department of Chemistry, Faculty of Science, Humanities and Education, Technical University of Liberec, Studentská 1402/2, 461 17, Liberec, Czech Republic; vera.jencova@tul.cz (V.J.)

* Correspondence: michal.rezanka@tul.cz (M.Ř.)

1. NMR spectra of 6-(pyrrol-3-yl)hexanoic acid

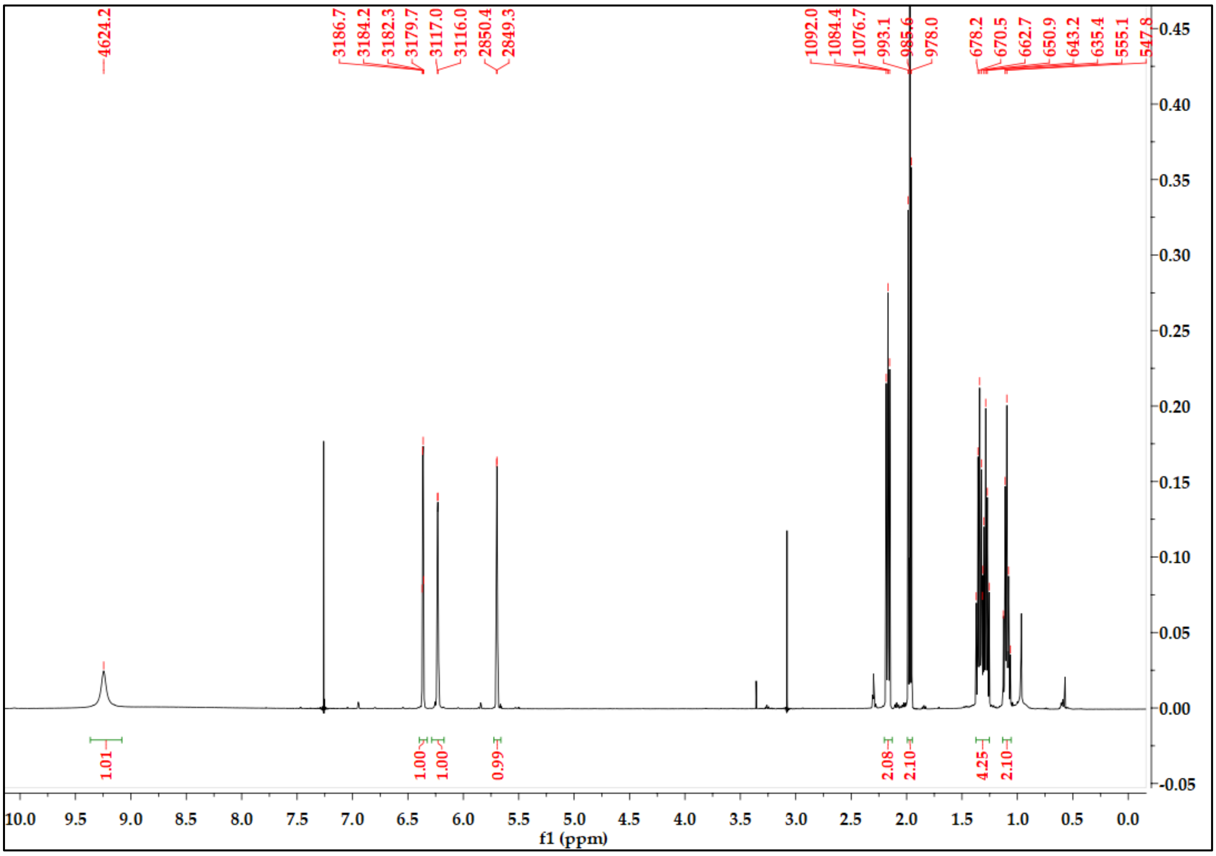

Figure S1. ^{1}H NMR spectrum ($CDCl_3$/MeOD, 298 K, 500 MHz).

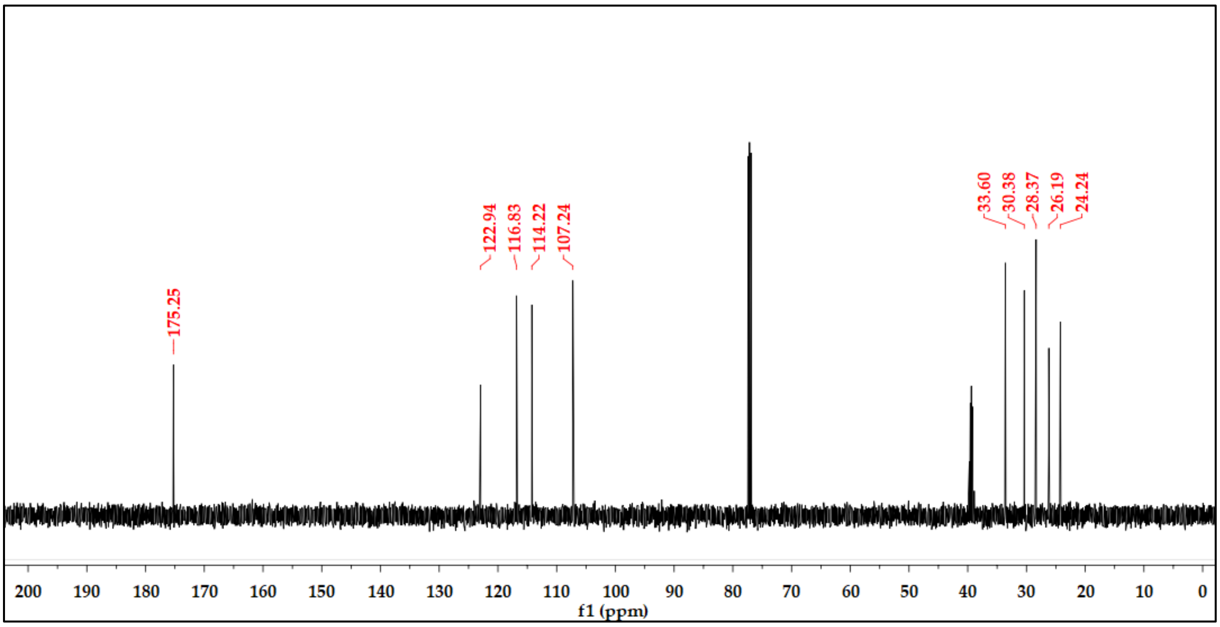

Figure S2. ^{13}C NMR spectrum ($CDCl_3$/MeOD, 298 K, 126 MHz).

2. Specific surface area measurement (BET)

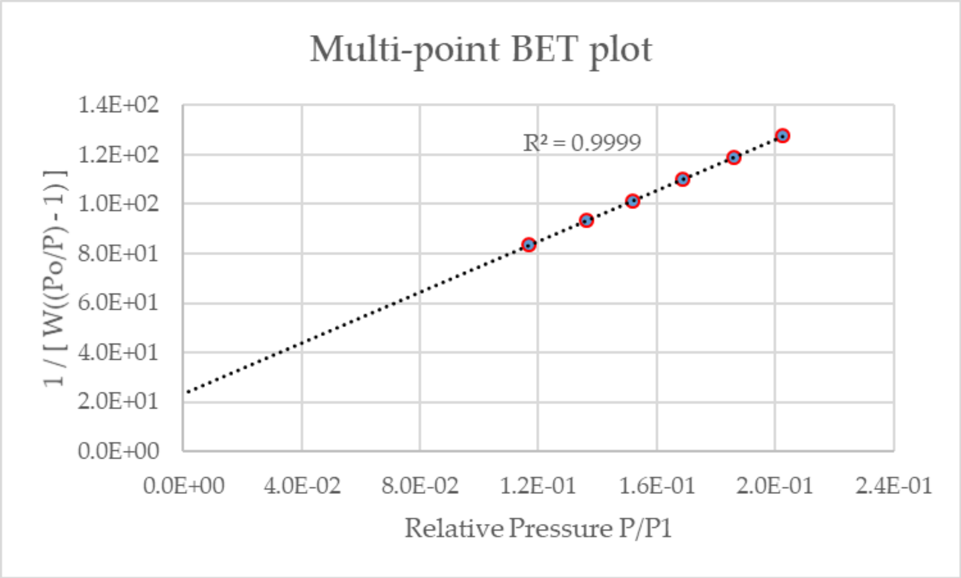

Figure S3. Multi-point BET plot.

3. Cell culturing

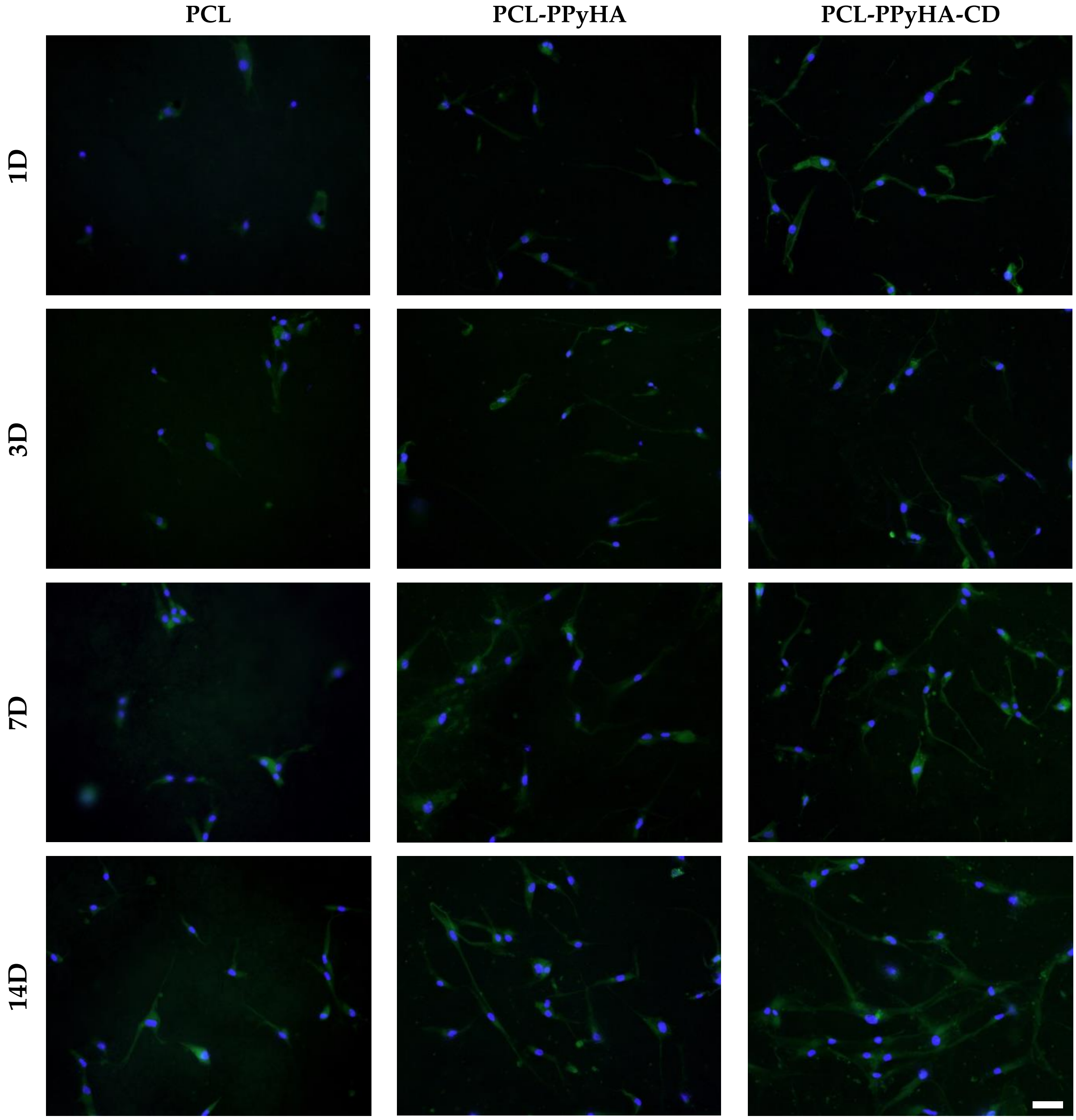

Figure S4. Fluorescent microscopy of cells seeded on PCL, PCL-PPyHA and PCL-PPyHA-CD scaffolds on days 1, 3, 7 and 14 after cell seeding. Staining: phalloidin-DAPI.
Scale bar: 50 μm.

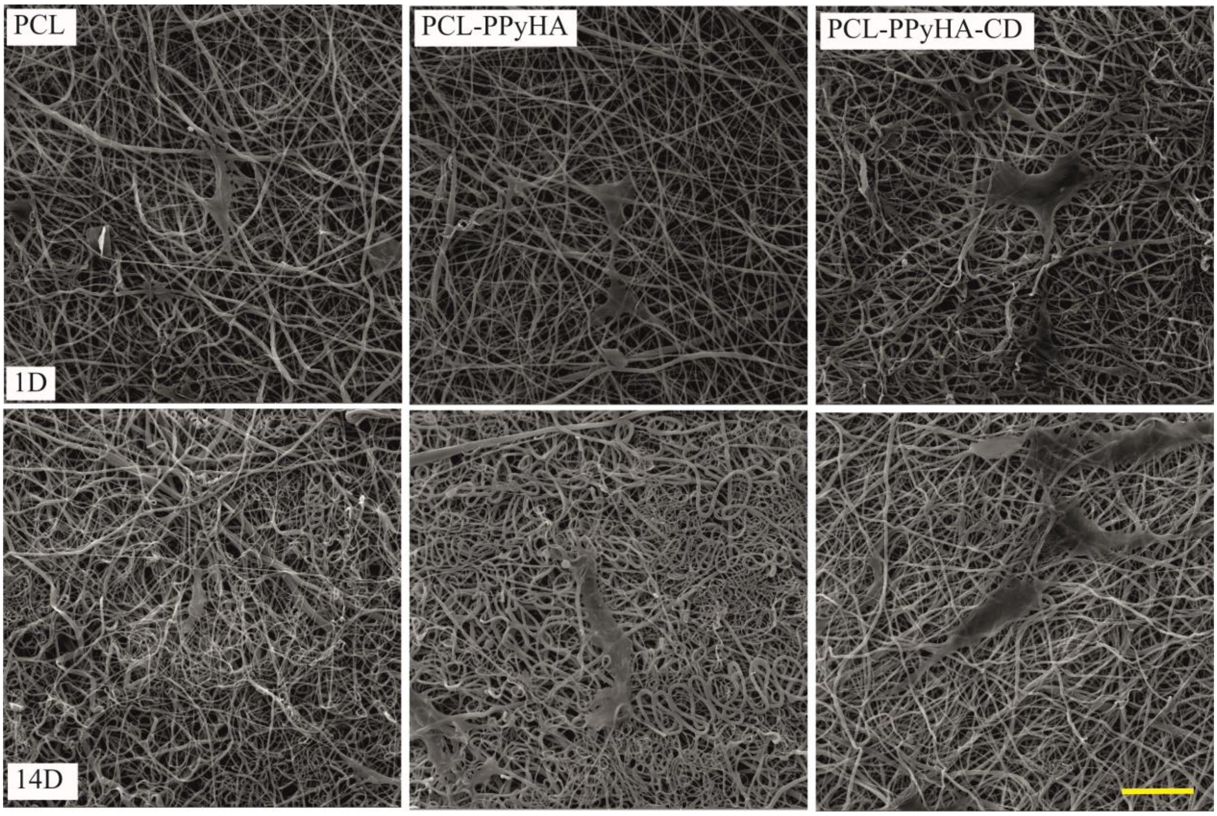

Figure S5. SEM images of the PCL, PCL-PPyHA and PCL-PPyHA-CD scaffolds on days 1 and 14 after cell seeding. Scale bar: 50 μm.
